# Postoperative Day 1 Mean Arterial Pressure and 30-Day Mortality or Day 7 Renal Replacement Therapy After Ventricular Assist Device Implantation

**DOI:** 10.7759/cureus.117178

**Published:** 2026-09-29

**Authors:** Kentaro Okamoto, Kimito Minami, Shigeki Fujitani, Muneyuki Takeuchi

**Affiliations:** 1 Department of Emergency and Critical Care Medicine, St. Marianna University School of Medicine, Kawasaki, JPN; 2 Department of Critical Care Medicine, National Cerebral and Cardiovascular Center, Suita, JPN

**Keywords:** biventricular assist device, mean arterial pressure, mechanical circulatory support, postoperative mortality, renal replacement therapy, right ventricular assist device, ventricular assist device

## Abstract

Background

The association between mean arterial pressure (MAP) early after ventricular assist device (VAD) implantation and short-term outcomes remains incompletely characterized. Our primary objective was to examine the association of postoperative day (POD) 1 MAP with death within 30 days or renal replacement therapy (RRT) on POD 7, accounting for initial support. Secondary exploratory objectives concerned other hemodynamic markers and subsequent renal and hepatic laboratory outcomes.

Methods

This single-center exploratory retrospective cohort included patients aged 18 years or older at an eligible index procedure between July 1, 2019, and March 31, 2025. Groups A-D represented initial VAD support strategies at the index procedure: (A) HeartMate 3 (Abbott Medical, Pleasanton, CA; HM3) alone; (B) HM3 with right ventricular assist device (RVAD) support; (C) extracorporeal left ventricular assist device (LVAD) alone; and (D) extracorporeal LVAD with RVAD support. Later support changes did not alter group assignment. The exposure was the median invasive MAP on POD 1. The composite outcome was death within 30 days or RRT on POD 7. Firth logistic regression included initial strategy and MAP centered within strategy. Seven hemodynamic markers were examined with Holm adjustment.

Results

The cohort comprised 156 patients (A, 92; B, five; C, 31; D, 28). The outcome was ascertainable in 146 patients, including 22 (15.1%) with events. Each 5-mmHg higher MAP was associated with lower odds of the outcome (odds ratio (OR), 0.217; 95% confidence interval (CI), 0.082-0.501; P < 0.001; Holm-adjusted P < 0.001). Mean perfusion pressure (MPP) was also associated with the outcome (OR per 5 mmHg, 0.412; 95% CI, 0.213-0.729). In a joint model, MAP remained associated (OR, 0.235; 95% CI, 0.085-0.553), whereas right atrial pressure did not. Sensitivity analyses showed the same direction of association. MAP was not clearly associated with subsequent creatinine or bilirubin summaries.

Conclusions

Lower invasive MAP on POD 1 was associated with early death or RRT on POD 7 after accounting for initial VAD support. These exploratory findings support further evaluation of MAP as an early risk marker; the effects and targets of interventions to increase MAP require further study.

## Introduction

Mechanical circulatory support includes durable implantable left ventricular assist devices (LVADs), extracorporeal LVADs, right ventricular assist devices (RVADs), and biventricular configurations. In Japan, extracorporeal support also provides a bridge to decisions regarding recovery or subsequent durable support [1]. These configurations are used in patients with different disease severity and therapeutic goals, making support-related differences in patient characteristics relevant to the evaluation of postoperative hemodynamics.

Blood-pressure management after ventricular assist device (VAD) implantation requires balancing organ perfusion against ventricular and pump loading. The 2023 International Society for Heart and Lung Transplantation (ISHLT) guideline recommends a mean arterial pressure (MAP) of 75-90 mmHg immediately after durable mechanical circulatory support implantation, with level C evidence for this early postoperative recommendation [2]. The American Heart Association statement emphasizes the interaction between arterial pressure and continuous-flow pump function [3]. Longitudinal LVAD cohorts have associated both low and high blood pressure with adverse outcomes [4].

The acute postoperative period differs from established support. Vasoplegia has been associated with adverse outcomes after LVAD implantation [5-7], while acute kidney injury and the requirement for renal replacement therapy (RRT) identify particularly vulnerable patients [8,9]. Mean perfusion pressure (MPP), approximated by MAP minus central venous or right atrial pressure (RAP), incorporates both arterial pressure and venous back pressure. Cardiac-surgery studies have examined absolute MPP, changes from baseline pressure, and venous congestion, with results that depend on the exposure definition and observation period [10-12]. Their applicability to extracorporeal and right-sided mechanical support remains uncertain.

The primary objective of this exploratory study was to examine the association between patient-level median invasive MAP on postoperative day (POD) 1 and the composite outcome of death within 30 days or RRT on POD 7, accounting for initial VAD support strategy. We evaluated the hypothesis that lower POD 1 MAP would be associated with higher odds of this outcome. Secondary exploratory objectives were to evaluate MPP and other pulmonary artery catheter-derived hemodynamic markers in relation to the composite outcome and to examine associations between POD 1 MAP and subsequent creatinine and bilirubin summaries. The MAP-focused hypothesis emerged during exploratory evaluation of multiple hemodynamic markers; this analysis should therefore be interpreted as hypothesis-generating rather than confirmatory.

## Materials and methods

Study design, setting, and ethics

This single-center exploratory retrospective cohort study used routinely recorded intensive care unit (ICU) and procedural data from the National Cerebral and Cardiovascular Center, Suita, Osaka, Japan. The eligibility window for index procedures was July 1, 2019, through March 31, 2025. The Research Ethics Committee of the National Cerebral and Cardiovascular Center approved the study (approval no. M30-026-11), waived individual written informed consent, and permitted an opt-out process. Reporting was guided by the STROBE statement and the RECORD extension for routinely collected health data [13,14].

Participants and initial VAD strategies

Patients aged 18 years or older at the index procedure were identified from procedure, device, and treatment records. Eligible procedures initiated one of the four VAD support configurations described below. Each patient was included once, at the first eligible procedure during the study period. Candidate procedures representing conversion or exchange of a pre-existing LVAD, or records in which an eligible device configuration could not be established, were excluded. Of 159 candidates assessed, three were excluded: one for conversion or exchange of a pre-existing LVAD and two because an eligible device configuration could not be established. Two clinicians reviewed the documented surgical and treatment course to determine initial support without referring to outcome information.

Patients were classified according to their initial support configuration: group A, HeartMate 3 (Abbott Medical, Pleasanton, CA; HM3) alone; group B, HM3 with RVAD support during the same operative episode; group C, extracorporeal LVAD alone; and group D, extracorporeal LVAD with RVAD support (extracorporeal biventricular assist device (BiVAD)). Classification was based on the documented procedural course rather than the procedure date alone. Subsequent RVAD insertion, conversion to HM3, pump exchange, or device removal did not change the initial group. The 156 eligible patients were classified once as group A (n = 92), group B (n = 5), group C (n = 31), or group D (n = 28).

Hemodynamic measurements and observation period

POD 1 was defined as the calendar day after the index procedure, from 00:00 to 23:59. Measurements on the procedure date were not included.

The primary exposure was the patient-level median of invasive MAP values recorded under the ACSYS item "ABP M" on POD 1. The primary-exposure dataset contained 3,970 MAP records. No duplicate records with the same patient, ICU stay, and recorded measurement time, and no special-value codes, were identified in these records. Valid extreme values were retained. The other markers were RAP, mean pulmonary artery pressure (PAPm), mixed venous oxygen saturation (SvO₂), continuous cardiac index (CCI), and continuous cardiac output (CCO), each summarized by its patient-level median. MPP was calculated as MAP minus RAP using measurements documented at the same time for the same patient and ICU admission. Pressure variables were expressed in mmHg, SvO₂ in percent, CCI in L/min/m², and CCO in L/min. CCI and CCO were recorded using the Edwards pulmonary artery catheter monitoring system (Edwards Lifesciences, Irvine, CA).

The paired analysis used 3,385 POD 1 MAP-RAP measurements; the analysis requiring simultaneous MAP, RAP, PAPm, and SvO₂ used 3,003 measurements. Measurements recorded at different times were not paired. Invalid measurements were not used, while valid extreme values were retained. Associations were reported per 0.5 L/min/m² higher CCI and per 1 L/min higher CCO.

Measurement and outcome availability are summarized in Appendix 1.

Source data comprised routinely recorded ICU, procedure/device, treatment, and laboratory records. The primary MAP exposure was extracted from the ACSYS "ABP M" item within the POD 1 calendar-day window. Postoperative windows were assigned relative to the index procedure date, and repeated hemodynamic measurements were summarized at the patient level. Paired hemodynamic analyses required the same patient, ICU stay, and recorded measurement time.

Outcomes

The composite outcome was death within 30 days of the index procedure or RRT on POD 7. Mortality was determined from documented death dates; survival through day 30 required clinical evidence on or after day 30, or a later documented death. POD 7 was the seventh calendar day after the index procedure. RRT on POD 7 was defined as any overlap between that calendar day and a documented interval of actual RRT delivery; a treatment order alone did not establish receipt of RRT. This included RRT begun earlier and continuing into POD 7, as well as RRT initiated on POD 7. The endpoint therefore described RRT receipt on that day, not initiation specifically on that day. The endpoint was based on actual RRT delivery without restriction to a particular modality. Individual modalities could be reliably classified from the treatment records, but the present endpoint pooled RRT rather than evaluating modality-specific outcomes. No patients in the cohort were receiving chronic maintenance dialysis.

A composite event was recorded when either component was confirmed. A non-event required both confirmed survival through day 30 and confirmed absence of RRT on POD 7. When the available records did not establish either an event or both conditions for a non-event, the outcome was classified as indeterminate. Ten composite outcomes were indeterminate and were excluded from the primary analysis. Sensitivity analyses assigned all ten first as non-events and then as events. A separate sensitivity analysis excluded patients with documented RRT on the day before the procedure; this restriction was not applied to the primary analysis.

Secondary analyses examined the maximum and median serum creatinine and total bilirubin concentrations on PODs 3-7, adjusted for the corresponding POD 1 value and initial VAD strategy.

Statistical analysis

Continuous variables are presented as medians (interquartile ranges) and categorical variables as counts and percentages. Firth bias-reduced logistic regression was used because of the small number of events and the absence of events in group B [15,16]. To account for differences in initial support, each model adjusted for the four-level support configuration as a categorical variable. The hemodynamic marker was centered by subtracting its mean within each strategy. MAP associations are reported per 5-mmHg increase, with profile penalized-likelihood 95% confidence intervals (CIs).

The Firth model maximized the binomial log-likelihood plus one-half of the log determinant of the Fisher information matrix. CIs were obtained by profiling the penalized likelihood. Marker centering used the strategy-specific mean among complete cases for each model. Models included an intercept, the centered marker, and indicators for groups B, C, and D, with group A as reference. Group-restricted models included the strategies present in the relevant subset.

The same approach was applied to seven markers: MAP, MPP, RAP, PAPm, SvO₂, CCI, and CCO. Holm adjustment addressed these seven comparisons. A model including MAP and RAP together examined the separate contributions of the two components of MPP. The MAP-focused analysis arose from exploratory evaluation of multiple hemodynamic markers.

Sensitivity analyses classified all indeterminate outcomes first as non-events and then as events; evaluated POD 7 RRT alone; used only contemporaneous MAP-RAP measurements or simultaneous MAP, RAP, PAPm, and SvO₂ measurements; excluded patients receiving RRT on the day before the procedure; and added POD 1 creatinine, age, and sex as covariates. Analyses were also repeated in selected strategy subsets. An additional Firth model replaced the four-level strategy variable with separate covariates for LVAD class (HM3 or extracorporeal LVAD) and initial RVAD use. This model used MAP centered at a common value of 70 mmHg and scaled per 5 mmHg. A quadratic MAP term assessed departure from linearity. Repeated analyses omitting individual patients, removing combinations of event patients, and using bootstrap samples assessed the influence of individual observations.

The alternative support-adjustment model included LVAD class (0, HM3; 1, extracorporeal LVAD) and initial RVAD use (0, absent; 1, present) as separate covariates, replacing the four-level strategy variable. MAP was entered as (MAP - 70)/5. This sensitivity model assumed additive effects of LVAD class and initial RVAD use on log odds.

Additional analyses examined the mean, time-weighted average over the observed measurement period, minimum, 25th percentile, and standard deviation of MAP and MPP. Time below selected pressure values was described as a proportion of the observed period. Laboratory outcomes were analyzed by linear regression with heteroscedasticity-consistent standard errors. Missing data were not imputed, and valid extreme values were retained. Tests were two-sided. P-values are reported to three decimal places; values below 0.001 are reported as P < 0.001.

The original analyses were performed using Python version 3.12.14 (Python Software Foundation, Wilmington, DE), NumPy version 2.3.5 (NumPy Developers, Python Software Foundation, Wilmington, DE), and SciPy version 1.17.0 (Travis Oliphant, Pearu Peterson, and Eric Jones, Rochester, MN). Firth bias-reduced logistic regression and profile penalized-likelihood calculations were implemented in custom, study-specific Python code using NumPy and SciPy. The primary model was reproduced, and the additional sensitivity analysis adjusting for LVAD class and initial RVAD use was performed, using Python version 3.12.14.

Distributional pressure analyses used contemporaneous MAP-RAP measurements and examined medians, means, time-weighted averages over the observed period, minima, lower quartiles, and standard deviations. Threshold analyses described the proportion of observed time below selected pressure values; they were not used to identify a treatment target. Linear models for creatinine and bilirubin on PODs 3-7 adjusted for the corresponding POD 1 value and initial strategy and used heteroscedasticity-consistent standard errors.

The broader project initially examined pulmonary artery catheter trajectories and MPP/RAP across support strategies. The MAP-focused hypothesis arose during exploratory evaluation of these hemodynamic markers. Null marker and laboratory-outcome findings are reported alongside the positive findings. Apparent and leave-one-out model-performance measures are exploratory and do not establish a validated clinical prediction model.

Data availability

The individual-level data underlying this study are not publicly available to protect patient confidentiality. Requests for access to de-identified data may be addressed to the corresponding author. Any sharing is subject to institutional authorization, applicable ethics requirements, and an appropriate data-use agreement.

## Results

Participants and outcome availability

The cohort included 156 patients: 92 in group A, five in group B, 31 in group C, and 28 in group D (Figure 1). Median age was 50 (41-59) years, and 101 patients (64.7%) were male. POD 1 invasive MAP was available in all 156 patients; MPP and RAP in 151; PAPm in 153; SvO₂ in 151; and CCI and CCO in 120 (Table 1).

**Figure 1:**
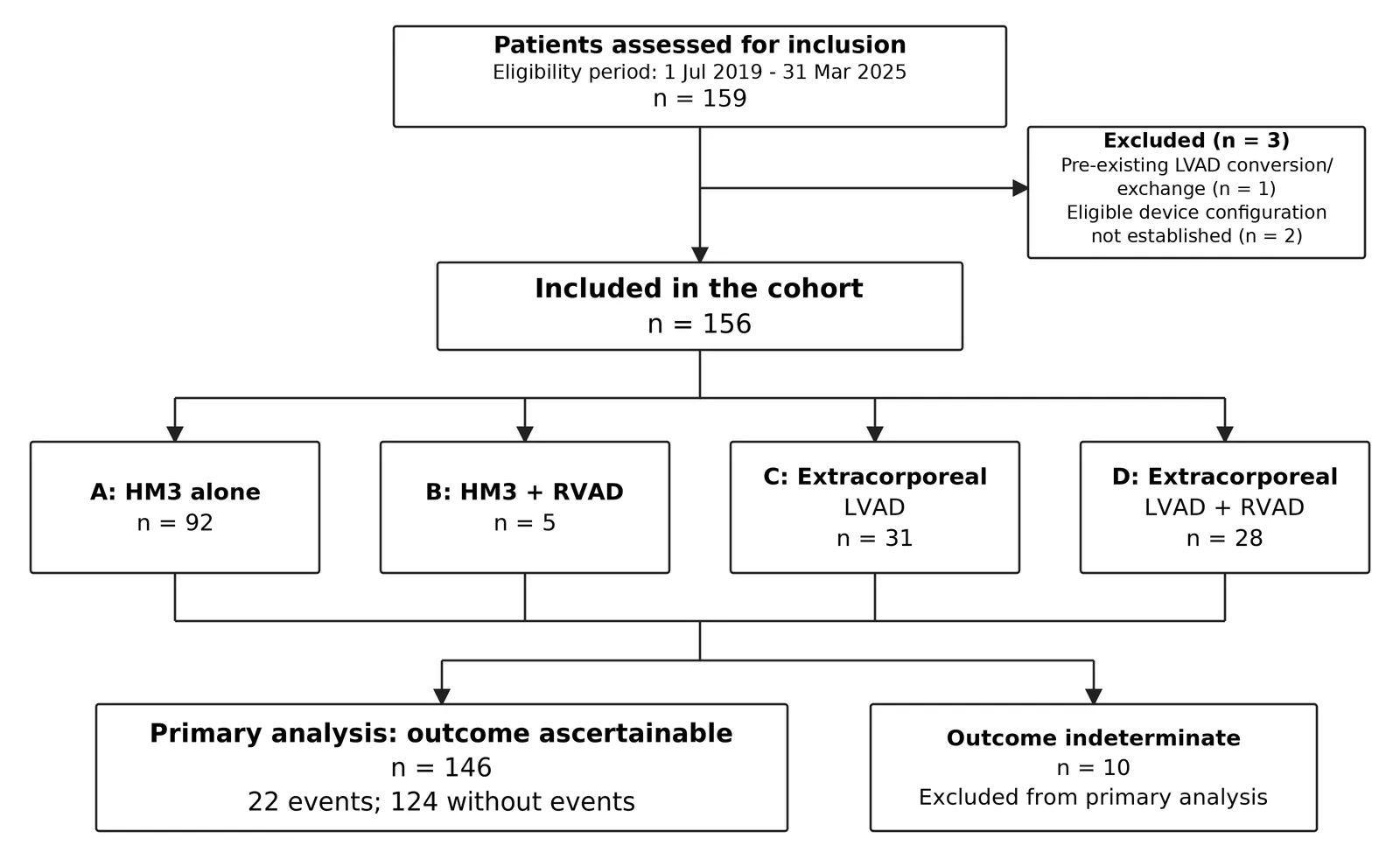
Patient selection and initial VAD strategies The eligibility window for index procedures was July 1, 2019, through March 31, 2025. Of 159 patients assessed for inclusion, 156 met the study criteria. Three candidates were excluded: one for conversion or exchange of a pre-existing LVAD and two because an eligible device configuration could not be established. The primary analysis included 146 patients with ascertainable composite outcomes. The 10 indeterminate outcomes were examined separately in sensitivity analyses. A, HM3 alone; B, HM3 + RVAD; C, extracorporeal LVAD; D, extracorporeal LVAD + RVAD (extracorporeal BiVAD). BiVAD, biventricular assist device; HM3, HeartMate 3; LVAD, left ventricular assist device; RVAD, right ventricular assist device; VAD, ventricular assist device.

**Table 1:** Patient and POD 1 characteristics by initial VAD strategy Data are medians (interquartile ranges) or n (%). Available-case n is shown when it differs from the column total. The study eligibility period is the window for identifying eligible index procedures, not the range of observed procedure dates. A, HM3 alone; B, HM3 + RVAD; C, extracorporeal LVAD; D, extracorporeal LVAD + RVAD (extracorporeal BiVAD). The composite-event percentages use patients with ascertainable outcomes as the denominator. Percentages for individual outcome components use the full group denominator; incomplete ascertainment should be considered. Outcome availability is detailed in Appendix 1. BiVAD, biventricular assist device; CCI, continuous cardiac index; CCO, continuous cardiac output; HM3, HeartMate 3; LVAD, left ventricular assist device; MAP, mean arterial pressure; MPP, mean perfusion pressure; PAPm, mean pulmonary artery pressure; POD, postoperative day; RAP, right atrial pressure; RRT, renal replacement therapy; RVAD, right ventricular assist device; SvO₂, mixed venous oxygen saturation; VAD, ventricular assist device.

Characteristic	A (n = 92)	B (n = 5)	C (n = 31)	D (n = 28)	Overall (n = 156)
Patients, n	92	5	31	28	156
Age, years	52.5 (44.0-59.0)	61.0 (59.0-63.0)	48.0 (37.0-53.5)	41.5 (36.5-53.0)	50.0 (41.0-59.0)
Male sex	60 (65.2%)	3 (60.0%)	20 (64.5%)	18 (64.3%)	101 (64.7%)
Study eligibility period	1 Jul 2019-31 Mar 2025	1 Jul 2019-31 Mar 2025	1 Jul 2019-31 Mar 2025	1 Jul 2019-31 Mar 2025	1 Jul 2019-31 Mar 2025
POD 1 invasive MAP, mmHg	69.5 (68.0-71.0)	68.5 (68.0-69.0)	71.0 (68.8-72.8)	70.0 (67.0-72.0)	69.5 (68.0-72.0)
POD 1 MPP, mmHg	59.0 (57.0-61.5) (n = 89)	58.0 (57.5-58.5)	59.0 (56.5-60.5)	59.0 (56.0-60.9) (n = 26)	59.0 (57.0-61.0) (n = 151)
POD 1 RAP, mmHg	10.0 (9.0-12.0) (n = 89)	10.5 (10.0-10.5)	12.0 (10.8-13.0)	11.0 (8.0-12.0) (n = 26)	10.5 (9.0-12.0) (n = 151)
POD 1 PAPm, mmHg	19.0 (16.0-22.0) (n = 91)	19.0 (18.0-21.5)	17.5 (16.0-19.8)	16.5 (14.2-19.0) (n = 26)	18.0 (16.0-21.5) (n = 153)
POD 1 SvO₂, %	74.0 (70.0-78.5) (n = 91)	78.0 (74.0-87.0)	77.0 (73.0-82.2)	87.5 (78.0-95.0) (n = 24)	76.0 (72.0-81.5) (n = 151)
POD 1 CCI, L/min/m²	2.92 (2.60-3.24) (n = 86)	1.55 (1.55-1.55) (n = 1)	2.80 (2.50-3.19) (n = 26)	3.35 (2.08-3.80) (n = 7)	2.90 (2.50-3.26) (n = 120)
POD 1 CCO, L/min	4.50 (3.90-5.38) (n = 86)	2.60 (2.60-2.60) (n = 1)	4.60 (4.22-5.66) (n = 26)	6.15 (3.92-7.10) (n = 7)	4.57 (3.90-5.40) (n = 120)
POD 1 creatinine, mg/dL	1.00 (0.73-1.26)	1.22 (0.79-1.40)	1.04 (0.81-1.81)	1.65 (0.97-2.87)	1.07 (0.77-1.48)
POD 1 total bilirubin, mg/dL	2.05 (1.60-3.00)	2.20 (1.90-2.35)	2.60 (2.12-4.41) (n = 30)	3.25 (2.10-5.43)	2.30 (1.70-3.35) (n = 155)
POD 1 hemoglobin, g/dL	10.9 (10.3-11.6)	8.8 (8.8-9.8)	10.9 (10.4-11.6)	10.4 (9.9-11.2)	10.8 (10.1-11.6)
POD 1 platelet count, ×10³/µL	120.0 (91.4-147.8)	100.5 (72.0-101.0)	105.5 (86.5-136.0)	80.2 (62.1-92.2)	104.0 (85.0-138.5)
Composite outcome ascertained	83 (90.2%)	4 (80.0%)	31 (100.0%)	28 (100.0%)	146 (93.6%)
30-day death	0 (0.0%)	0 (0.0%)	2 (6.5%)	3 (10.7%)	5 (3.2%)
RRT on POD 7	3 (3.3%)	0 (0.0%)	3 (9.7%)	12 (42.9%)	18 (11.5%)
Composite event, among ascertained	3 (3.6%)	0 (0.0%)	5 (16.1%)	14 (50.0%)	22 (15.1%)

CCI and CCO were each available in 120 of 156 patients (76.9%) and missing in 36 (23.1%). Availability differed by initial strategy: 86/92 (93.5%) in group A, 1/5 (20.0%) in group B, 26/31 (83.9%) in group C, and 7/28 (25.0%) in group D. The CCI and CCO models each included 112 patients with 11 events, compared with 146 patients with 22 events in the primary MAP model (Table 1 and Table 2).

**Table 2:** Strategy-adjusted associations of POD 1 hemodynamic markers Each marker was centered within strategy and entered into a separate Firth model with strategy as a categorical variable. Intervals and P values use the profile penalized likelihood. Holm correction covers the seven markers. CCI, continuous cardiac index; CCO, continuous cardiac output; CI, confidence interval; MAP, mean arterial pressure; MPP, mean perfusion pressure; OR, odds ratio; PAPm, mean pulmonary artery pressure; POD, postoperative day; RAP, right atrial pressure; SvO₂, mixed venous oxygen saturation.

Marker	Effect scale	n/events	OR	95% CI	Raw P	Holm P
Invasive MAP	+5 mmHg	146/22	0.217	0.082-0.501	<0.001	<0.001
MPP = MAP - RAP	+5 mmHg	142/21	0.412	0.213-0.729	0.002	0.011
RAP	+2 mmHg	142/21	1.214	0.808-1.857	0.353	1
PAPm	+5 mmHg	143/21	0.662	0.325-1.248	0.209	1
SvO₂	+5 percentage points	141/21	0.984	0.710-1.354	0.92	1
CCI	+0.5 L/min/m²	112/11	0.75	0.456-1.189	0.223	1
CCO	+1 L/min	112/11	0.815	0.478-1.331	0.418	1

The composite outcome was ascertainable in 146 patients (93.6%), of whom 22 (15.1%) experienced an event: 17 had RRT without documented death within 30 days, four died without RRT on POD 7, and one had both components. Outcomes were indeterminate in 10 patients. Among patients with ascertainable outcomes, events occurred in three of 83 (3.6%) in group A, zero of four (0.0%) in group B, five of 31 (16.1%) in group C, and 14 of 28 (50.0%) in group D (Table 1).

Associations of hemodynamic markers with the composite outcome

Among patients with ascertainable outcomes, median POD 1 MAP was 68.0 (65.6-69.9) mmHg in those with events and 70.0 (68.0-72.0) mmHg in those without. In the strategy-adjusted model, each 5-mmHg higher MAP was associated with lower odds of the outcome (odds ratio (OR), 0.217; 95% CI, 0.082-0.501; P < 0.001; Holm-adjusted P < 0.001). MPP was also associated with lower odds (OR per 5 mmHg, 0.412; 95% CI, 0.213-0.729; Holm-adjusted P = 0.011). RAP, PAPm, SvO₂, CCI, and CCO showed no clear associations (Table 2; Figure 2).

**Figure 2:**
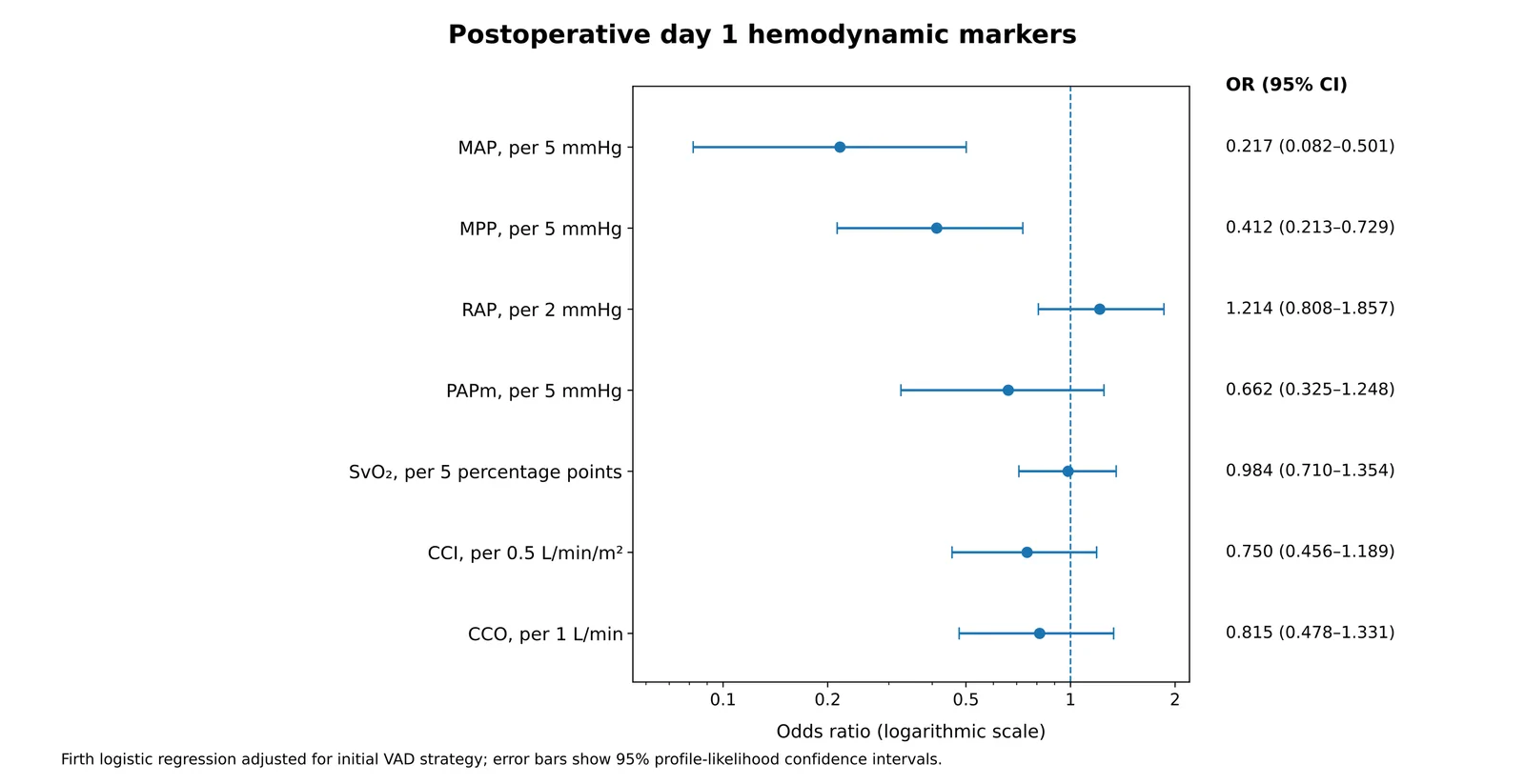
Associations of POD 1 hemodynamic markers with death within 30 days or RRT on POD 7 Separate Firth models included initial strategy and the marker centered within strategy. Horizontal lines show 95% confidence intervals. Effects are reported per 0.5 L/min/m² higher CCI and per 1 L/min higher CCO. CCI, continuous cardiac index; CCO, continuous cardiac output; CI, confidence interval; MAP, mean arterial pressure; MPP, mean perfusion pressure; OR, odds ratio; PAPm, mean pulmonary artery pressure; POD, postoperative day; RAP, right atrial pressure; RRT, renal replacement therapy; SvO₂, mixed venous oxygen saturation; VAD, ventricular assist device.

The estimates for MAP and RAP entered jointly are presented in Appendix 6.

In the joint MAP-RAP model, MAP remained associated with the outcome (OR per 5 mmHg, 0.235; 95% CI, 0.085-0.553; P < 0.001), whereas RAP did not (OR per 2 mmHg, 1.009; 95% CI, 0.657-1.575; P = 0.968). MAP correlated with MPP (r = 0.758), but its correlation with RAP was negligible (r = -0.034). These results suggest that the observed MPP association mainly reflected its arterial-pressure component in this cohort.

Sensitivity and influence analyses

The direction of the MAP association was maintained across sensitivity analyses (Table 3; Figure 3). With LVAD class and initial RVAD use entered as separate covariates, the OR per 5-mmHg higher MAP was 0.211 (95% CI, 0.079-0.489; P < 0.001).

**Table 3:** Sensitivity analyses for POD 1 invasive MAP All estimates are per 5-mmHg higher MAP. Initial support was adjusted using four strategies, except in the LVAD class and initial RVAD model, which entered these two factors separately instead of the four-level variable. CI, confidence interval; HM3, HeartMate 3; LVAD, left ventricular assist device; MAP, mean arterial pressure; OR, odds ratio; PAPm, mean pulmonary artery pressure; POD, postoperative day; RAP, right atrial pressure; RRT, renal replacement therapy; RVAD, right ventricular assist device; SvO₂, mixed venous oxygen saturation.

Analysis	n/events	OR per 5 mmHg	95% CI	P
Primary analysis	146/22	0.217	0.082-0.501	<0.001
Adjusted for LVAD class and initial RVAD	146/22	0.211	0.079-0.489	<0.001
Indeterminate outcomes assigned no event	156/22	0.214	0.081-0.495	<0.001
Indeterminate outcomes assigned events	156/32	0.377	0.183-0.708	0.002
RRT on POD 7 only	152/18	0.415	0.178-0.860	0.016
Simultaneous MAP, RAP, PAPm, and SvO₂	139/20	0.27	0.112-0.582	<0.001
MAP from contemporaneous MAP-RAP measurements	142/21	0.252	0.100-0.568	<0.001
Exclude RRT on the day before the procedure	145/21	0.215	0.081-0.496	<0.001
Adjusted for POD 1 creatinine	146/22	0.256	0.095-0.597	<0.001
Adjusted for age and sex	146/22	0.22	0.083-0.508	<0.001
Adjusted for creatinine, age, and sex	146/22	0.241	0.085-0.596	<0.001

**Figure 3:**
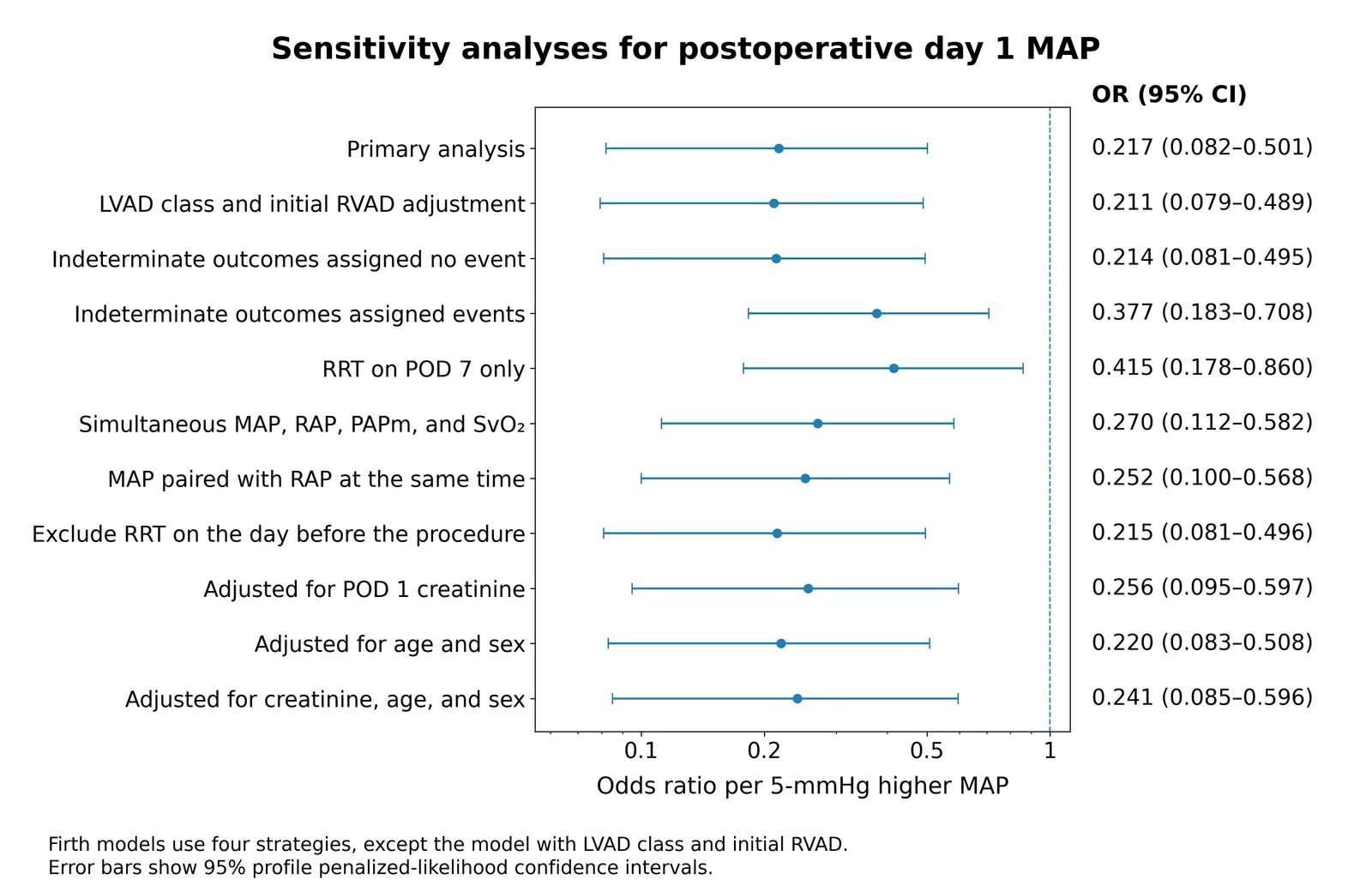
Sensitivity analyses of the association between POD 1 invasive MAP and the composite outcome Estimates are per 5-mmHg higher MAP. The LVAD class and initial RVAD model entered these two factors instead of the four-level strategy variable. The vertical dashed line indicates an OR of 1.  CI, confidence interval; LVAD, left ventricular assist device; MAP, mean arterial pressure; OR, odds ratio; POD, postoperative day; RAP, right atrial pressure; RRT, renal replacement therapy; RVAD, right ventricular assist device; PAPm, mean pulmonary artery pressure; SvO₂, mixed venous oxygen saturation.

The same 146 patients and 22 events contributed to both models. The OR per 5-mmHg increase in MAP was 0.211 (95% CI, 0.079-0.489; profile penalized-likelihood P < 0.001).

Assigning all 10 indeterminate outcomes as events yielded an OR of 0.377 (95% CI, 0.183-0.708). The OR was 0.415 (95% CI, 0.178-0.860) for RRT on POD 7 alone, 0.252 (95% CI, 0.100-0.568) when MAP was restricted to contemporaneous MAP-RAP measurements, and 0.241 (95% CI, 0.085-0.596) after additional adjustment for POD 1 creatinine, age, and sex.

The primary analysis used each patient’s median of all valid POD 1 invasive MAP measurements and included 146 patients with 22 events (OR per 5-mmHg higher MAP, 0.217; 95% CI, 0.082-0.501). In the contemporaneous MAP-RAP analysis, MAP medians were calculated using only readings with RAP recorded at the same time; the analysis included 142 patients with 21 events (OR, 0.252; 95% CI, 0.100-0.568). These analyses therefore differed both in the included patients and in the MAP readings contributing to patient-level medians. The estimates should not be interpreted as results from identical analytic samples.

The estimates obtained after restricting or omitting selected support strategies are presented in Appendix 2.

Estimates had the same direction in groups A, C, and D and in the examined two-group combinations (Appendix 2). The group C estimate was imprecise, with only five events. Leave-one-patient-out ORs ranged from 0.187 to 0.279; the median OR after removing every combination of three event patients was 0.216. Adding a quadratic MAP term did not improve model fit (P = 0.737). Figure 4 displays the strategy-standardized model-predicted probability across the central 95% of observed MAP values.

**Figure 4:**
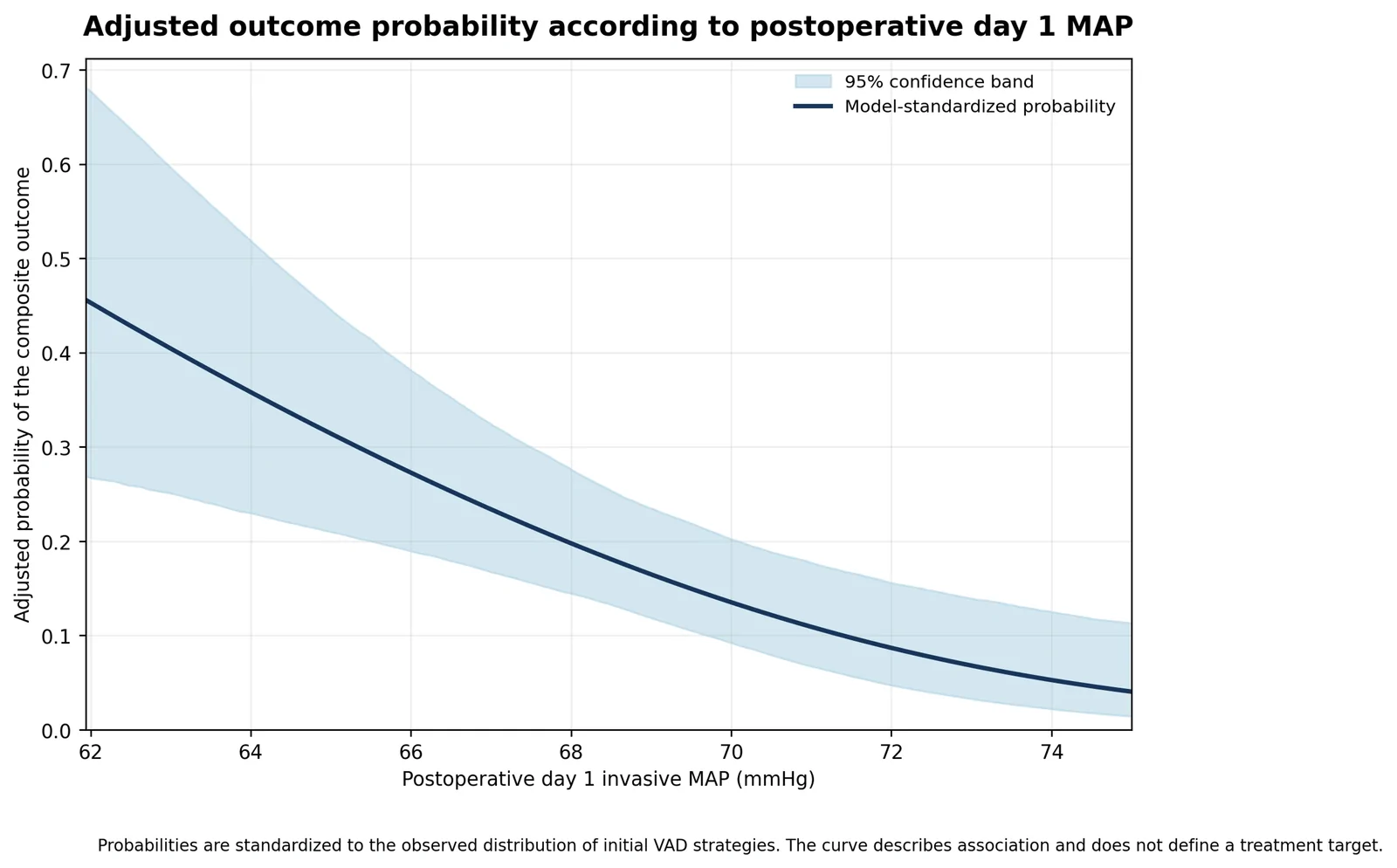
Strategy-standardized predicted probability of the composite outcome across the central 95% of observed POD 1 MAP values The shaded area represents the 95% confidence interval. The curve describes an association and does not establish a treatment target. MAP, mean arterial pressure; POD, postoperative day.

The original quadratic check added the square of the within-strategy-centered MAP term (P = 0.737). A check using MAP centered at the common value of 70 mmHg also showed no evidence of a quadratic term (P = 0.719).

Appendix 8 displays the group-restricted and leave-one-strategy-out MAP estimates.

Additional pressure and laboratory analyses

Associations for alternative distributional summaries of MAP and MPP are presented in Appendix 3.

Associations for the proportion of observed time below selected pressure values are shown in Appendix 4.

Median, mean, time-weighted average, and 25th-percentile MAP calculated from contemporaneous MAP-RAP measurements showed similar associations; the association for minimum MAP was weaker. More observed time below 65 or 70 mmHg was associated with higher odds of the outcome (Appendix 3 and Appendix 4). There were no clear associations between POD 1 MAP and the maximum or median creatinine or total bilirubin on PODs 3-7 after adjustment for the corresponding POD 1 laboratory value and initial strategy (Appendix 5).

The renal and hepatic laboratory outcome analyses are summarized in Appendix 5.

Among the 146 patients with ascertainable composite outcomes, the observed event proportions were 4/9 (44.4%) for POD 1 MAP <65 mmHg, 12/66 (18.2%) for 65 to <70 mmHg, 6/65 (9.2%) for 70 to <75 mmHg, and 0/6 (0.0%) for ≥75 mmHg (Appendix 9). These are unadjusted descriptive proportions, not estimates of an intervention effect or evidence for a treatment threshold.

Apparent and leave-one-out model-performance summaries are presented in Appendix 7.

## Discussion

Lower invasive MAP on POD 1 was associated with death within 30 days or RRT on POD 7 after adjustment for initial VAD support. MPP was also associated with the outcome, and MAP retained its association in the joint MAP-RAP model. The direction was consistent across sensitivity analyses, with a similar estimate when LVAD class and initial RVAD use were entered as separate covariates.

The INTERMACS analysis by Cowger and colleagues included 16,155 operative survivors and associated low and very high average blood pressures during LVAD support with adverse outcomes [4]. Our study focused on POD 1, a different observation period from that assessment of average pressure during longer-term support. ISHLT recommends a MAP of 75-90 mmHg immediately after implantation, based on level C evidence [2]. The median MAP of 69.5 mmHg in our cohort describes pressures achieved during care; evaluating the appropriateness of treatment targets requires intervention studies. The afterload dependence of continuous-flow pumps also warrants consideration of the relationship between arterial pressure, forward flow, and oxygen delivery [3].

Agreement between noninvasive measurements and arterial-line MAP is limited in HM3 recipients [17]. We restricted the analysis to arterial-line MAP. The patient-level POD 1 median characterizes the prevailing pressure over that day. Similar associations for the mean, time-weighted average, and lower quartile support an association with the broader pressure distribution.

Postimplantation vasoplegia is a plausible explanation for the association between low MAP and adverse outcomes. de Waal et al. and Tecson et al. and their colleagues linked vasoplegia after LVAD implantation to adverse postoperative outcomes [5,6]. In a 600-patient cohort, Lamba and colleagues found higher 30-day mortality with prolonged than with transient vasoplegia (16.7% versus 5.8%) [7]. Their definition incorporated vasoactive support and maintained cardiac index, illustrating that low pressure can coexist with preserved measured flow. In our study, low MAP may similarly reflect reduced vascular tone, inadequate effective circulating volume, bleeding, impaired cardiac or device flow, or combinations of these processes. Without detailed vasoactive doses, pump-flow measurements, and treatment goals, it is not possible to distinguish persistent vasodilation from other forms of circulatory failure. Low MAP in this cohort may reflect residual circulatory instability arising from these processes.

Hu and colleagues associated the duration of reduced MPP during cardiopulmonary bypass, relative to baseline, with subsequent acute kidney injury [10]. By contrast, the multicenter retrospective study by Kotani and colleagues found no association between relative postoperative MPP deficit and acute kidney injury progression, although lower absolute MPP and higher central venous pressure were associated in exploratory analyses [11]. That study excluded patients receiving mechanical circulatory support, further limiting direct extrapolation. Demirjian and colleagues also reported an interaction between MAP and central venous pressure in a large cardiac-surgery cohort [12]. Together, these studies support evaluating arterial and venous pressures jointly while considering the observation period and population.

MAP retained its association in the joint model, whereas RAP showed no clear association. This pattern is consistent with a possible contribution of arterial pressure to the MPP association, but does not establish a difference in the contributions of MAP and RAP. In RECOVER RIGHT, central venous pressure decreased after initiation of Impella RP support [18]. The PEEP-test study also demonstrated changes in cardiac output with ventilator settings that depended on volume responsiveness [19]. These studies provide physiological context, although their populations and interventions differ from those studied here. The possibility that support configuration and treatment modify the interpretation of RAP and MPP warrants consideration, but differences between univentricular and biventricular support were not directly tested in this study.

PAPm, SvO₂, CCI, and CCO showed no clear associations. Availability was unequal: CCI and CCO were available in only one patient in group B and seven in group D. The substantial and strategy-dependent missingness may have introduced selection related to measurement availability. Moreover, the CCI/CCO models included only 112 patients with 11 events, rather than the 146 patients with 22 events in the primary MAP model. The null CCI/CCO findings therefore do not establish absence of prognostic relevance, and differences in statistical significance cannot establish superiority of MAP over these markers. Exploratory model-performance summaries are provided in Appendix 7.

Muslem and colleagues associated acute kidney injury after LVAD implantation with adverse survival [8], and Asleh and colleagues identified early RRT as a marker of high risk with important preoperative renal and hemodynamic determinants [9]. In our cohort, 18 of 22 composite events included RRT on POD 7. Thus, RRT accounted for most composite events, although this does not establish its contribution to the observed MAP association. In critically ill patients with acute kidney injury, STARRT-AKI showed that the RRT initiation strategy affected the proportion of patients receiving RRT [20]. RRT on POD 7 may therefore reflect clinical decisions as well as persistent renal dysfunction; STARRT-AKI did not specifically evaluate this endpoint in VAD recipients. Only five deaths occurred, limiting separate assessment of the mortality association.

Subsequent creatinine and bilirubin summaries showed no clear associations with MAP. During RRT, extracorporeal solute clearance complicates assessment of native kidney function using serum creatinine [21]. Fluid accumulation can also dilute serum creatinine and delay recognition of acute kidney injury [22]. These considerations concern creatinine and do not directly explain the bilirubin findings. In this cohort, survival and repeated-sample availability may also have influenced laboratory summaries. Given these limitations, the short observation period, and the sample size, the absence of clear laboratory associations neither excludes renal or hepatic injury nor establishes the mechanism linking MAP to the composite outcome.

Prospective evaluation is needed to translate the observed MAP association into a treatment strategy. The PrevHemAKI randomized trial did not show a reduction in cardiac-surgery-associated acute kidney injury with individualized MPP targets, but also did not achieve separation in postoperative MAP or MPP between groups [23]. This limits inference about the effect of achieving higher pressures. Future VAD studies should record baseline renal status, serial arterial and venous pressures, vasoactive doses, pump settings and flow, bleeding, and reasons for RRT initiation and withdrawal to evaluate strategy-specific or individualized blood-pressure interventions.

Limitations

This single-center retrospective study is exploratory. Adjustment for seven comparisons does not eliminate uncertainty arising from data-driven hypothesis formation or multiple subsequent analyses. Only 22 composite events occurred, limiting precision despite the use of Firth regression. Group B included five patients, with four ascertainable outcomes and no events. There were only three events among all HM3 recipients, limiting assessment of configuration-specific associations and interactions. Independent confirmation is required. The single-center setting and limited sample size constrain generalizability. Confirmation in larger, multicenter cohorts encompassing a broader range of patients and care settings is needed before the findings can inform treatment recommendations.

Initial support strategy was closely related to clinical indication, disease severity, treatment era, and intended pathway, so confounding by indication may persist after four-strategy adjustment. Detailed vasoactive medication doses, clinician-selected MAP targets, device flow, ventilator settings, bleeding, and precise operative and cardiopulmonary-bypass times were unavailable. The same observed MAP may therefore reflect different underlying states and treatment intensities. The calendar-day definition produced different intervals from procedure completion, and a daily median cannot fully characterize the depth and duration of hypotension.

The composite endpoint combines outcomes at different time points and was dominated by RRT. RRT use is influenced by baseline renal injury and clinical decisions. Although no patients were receiving chronic maintenance dialysis, the POD 7 endpoint could include continuation of acute renal support begun before implantation as well as postoperative initiation; it was not an incident-AKI endpoint. Mortality events were too few to support a separate conclusion about survival. Ten outcomes remained indeterminate; the extreme-case analyses retained the direction of association but do not rule out bias from incomplete follow-up. Laboratory analyses additionally depended on survival, sampling, and RRT exposure.

Availability differed substantially across hemodynamic markers, especially CCI and CCO in right-sided support, limiting direct comparisons between markers and strategies. The number of events and observed MAP range were insufficient to evaluate nonlinear relationships or optimal MAP values reliably. The model-predicted probabilities have not been externally validated.

## Conclusions

Lower invasive MAP on POD 1 was associated with early death or RRT on POD 7 after adjustment for initial VAD support. These exploratory findings support further evaluation of POD 1 MAP as an early risk marker. Larger, multicenter prospective studies are needed to determine whether interventions to increase MAP improve outcomes and to establish appropriate treatment targets.

## Figures and Tables

**Figure 5:**
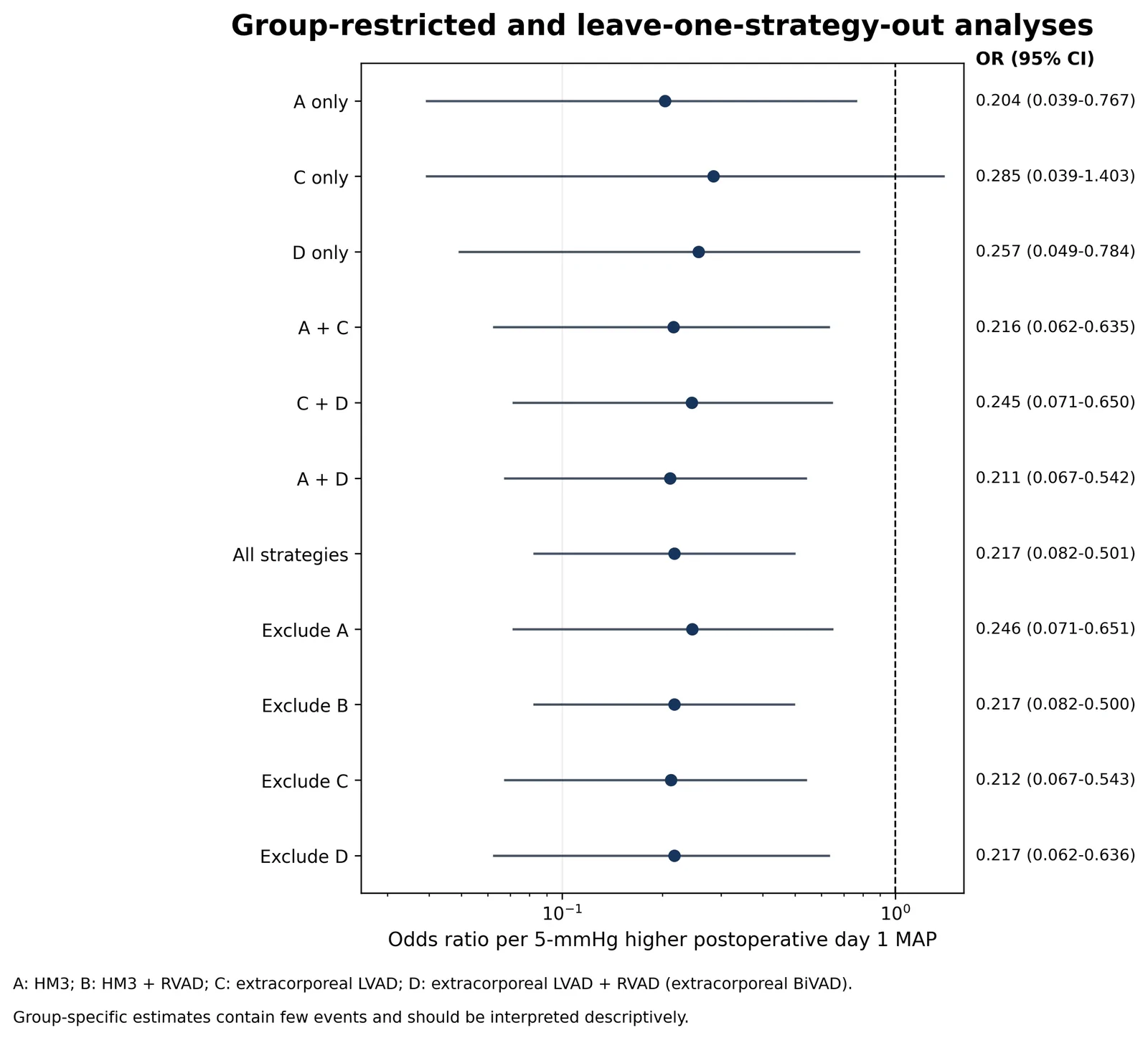
Group-restricted and leave-one-strategy-out estimates for POD 1 MAP Group-restricted and leave-one-strategy-out estimates for POD 1 MAP, per 5-mmHg increase. Group-specific estimates have few events and should be interpreted descriptively. A, HM3 alone; B, HM3 + RVAD; C, extracorporeal LVAD; D, extracorporeal LVAD + RVAD (extracorporeal BiVAD). BiVAD, biventricular assist device; CI, confidence interval; HM3, HeartMate 3; LVAD, left ventricular assist device; MAP, mean arterial pressure; OR, odds ratio; POD, postoperative day; RVAD, right ventricular assist device.

**Figure 6:**
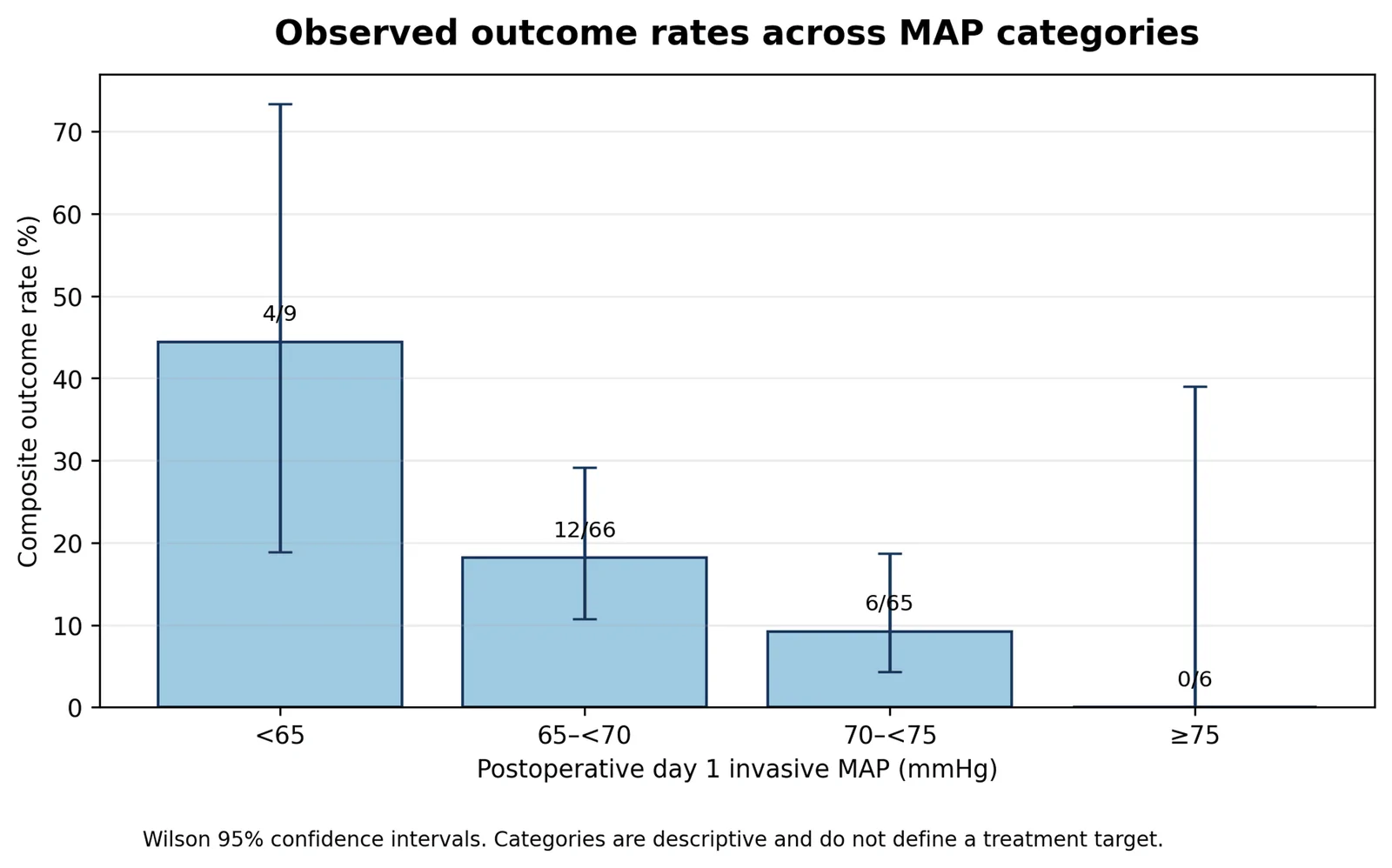
Observed composite-event rates across descriptive POD 1 MAP categories Observed composite-event rates across descriptive POD 1 MAP categories. Error bars are Wilson 95% confidence intervals. Categories do not define a treatment target. MAP, mean arterial pressure; POD, postoperative day.

## Appendices

Appendix 1

**Table 4:** Measurement and outcome availability Availability describes whether each variable could be evaluated, not necessarily eligibility for every complete-case model. A, HM3 alone; B, HM3 + RVAD; C, extracorporeal LVAD; D, extracorporeal LVAD + RVAD (extracorporeal BiVAD). BiVAD, biventricular assist device; CCI, continuous cardiac index; CCO, continuous cardiac output; HM3, HeartMate 3; LVAD, left ventricular assist device; MAP, mean arterial pressure; MPP, mean perfusion pressure; PAPm, mean pulmonary artery pressure; POD, postoperative day; RAP, right atrial pressure; RRT, renal replacement therapy; RVAD, right ventricular assist device; SvO₂, mixed venous oxygen saturation; VAD, ventricular assist device.

Variable	Total	Observed	Missing	Observed %
POD 1 invasive MAP	156	156	0	100.00%
POD 1 MPP	156	151	5	96.80%
POD 1 RAP	156	151	5	96.80%
POD 1 PAPm	156	153	3	98.10%
POD 1 SvO₂	156	151	5	96.80%
POD 1 CCI	156	120	36	76.90%
POD 1 CCO	156	120	36	76.90%
Simultaneous MAP/RAP/PAPm/SvO₂	156	148	8	94.90%
POD 1 creatinine	156	156	0	100.00%
POD 1 total bilirubin	156	155	1	99.40%
30-day vital status	156	146	10	93.60%
POD 7 RRT status	156	152	4	97.40%
Composite outcome	156	146	10	93.60%

Appendix 2

**Table 5:** Analyses restricted to or omitting selected strategies Estimates are per 5-mmHg higher MAP. Multi-strategy models account for the included strategies. Single-strategy estimates are descriptive because of the small numbers of events. A, HM3 alone; B, HM3 + RVAD; C, extracorporeal LVAD; D, extracorporeal LVAD + RVAD (extracorporeal BiVAD). BiVAD, biventricular assist device; CI, confidence interval; HM3, HeartMate 3; LVAD, left ventricular assist device; MAP, mean arterial pressure; OR, odds ratio; RVAD, right ventricular assist device.

Analysis set	Groups	n/events	OR per 5 mmHg	95% CI	P
A only	A	83/3	0.204	0.039-0.767	0.021
C only	C	31/5	0.285	0.039-1.403	0.132
D only	D	28/14	0.257	0.049-0.784	0.011
A + C	A+C	114/8	0.216	0.062-0.635	0.006
C + D	C+D	59/19	0.245	0.071-0.650	0.002
A + D	A+D	111/17	0.211	0.067-0.542	<0.001
All strategies	A+B+C+D	146/22	0.217	0.082-0.501	<0.001
Exclude A	B+C+D	63/19	0.246	0.071-0.651	0.002
Exclude B	A+C+D	142/22	0.217	0.082-0.500	<0.001
Exclude C	A+B+D	115/17	0.212	0.067-0.543	<0.001
Exclude D	A+B+C	118/8	0.217	0.062-0.636	0.006

Appendix 3

**Table 6:** Distributional summaries of MAP and MPP Effects are per 5-mmHg increase except standard deviation (SD), which is per 2 mmHg. Analyses use the contemporaneous MAP-RAP subset; therefore, the median-MAP estimate differs from the unrestricted primary analysis. CI, confidence interval; MAP, mean arterial pressure; MPP, mean perfusion pressure; OR, odds ratio; RAP, right atrial pressure; SD, standard deviation.

Summary	n/events	OR	95% CI	P
MAP median	142/21	0.252	0.100-0.568	<0.001
MAP mean	142/21	0.248	0.090-0.613	0.001
MAP time-weighted average	142/21	0.253	0.097-0.597	0.001
MAP minimum	142/21	0.655	0.439-0.984	0.042
MAP 25th percentile	142/21	0.313	0.136-0.654	0.001
MAP SD (per 2 mmHg)	142/21	1.293	0.784-2.073	0.299
MPP median	142/21	0.412	0.213-0.729	0.002
MPP mean	142/21	0.524	0.287-0.903	0.019
MPP time-weighted average	142/21	0.499	0.274-0.863	0.012
MPP minimum	142/21	0.99	0.920-1.095	0.812
MPP 25th percentile	142/21	0.503	0.277-0.864	0.011
MPP SD (per 2 mmHg)	142/21	1.01	0.809-1.147	0.902

Appendix 4

**Table 7:** Observed time below selected pressure values Pressure cutoffs are in mmHg. Effects are per 10-percentage-point increase in observed time below the listed value. Median and upper-quartile (Q3) percentages describe the exposure distribution. These analyses do not establish a therapeutic threshold. CI, confidence interval; MAP, mean arterial pressure; MPP, mean perfusion pressure; OR, odds ratio; Q3, upper quartile.

Exposure	n/events	OR per 10 percentage points	95% CI	P	Median %	Q3 %
MAP <60	142/21	1.695	1.154-2.764	0.007	0	1.1
MAP <65	142/21	1.435	1.116-1.880	0.005	10.6	22.4
MAP <70	142/21	1.367	1.089-1.773	0.006	48	66.1
MAP <75	142/21	1.999	1.295-3.458	<0.001	87.2	95.8
MPP <50	142/21	1.553	1.119-2.264	0.008	4.1	11.3
MPP <55	142/21	1.279	1.027-1.611	0.028	17.3	31
MPP <60	142/21	1.213	0.998-1.506	0.052	56	73.4

Appendix 5

**Table 8:** Continuous renal and hepatic outcomes Coefficients are changes in mg/dL per 5-mmHg higher MAP. Models adjust for initial strategy and the corresponding POD 1 laboratory value. No clear associations were identified. CI, confidence interval; MAP, mean arterial pressure; POD, postoperative day.

Outcome	n	Coefficient per 5-mmHg higher MAP	95% CI	P
PODs 3-7 creatinine maximum	125	-0.065	-0.214-0.084	0.393
PODs 3-7 creatinine median	125	-0.032	-0.154-0.089	0.602
PODs 3-7 total bilirubin maximum	124	-0.683	-2.214-0.848	0.382
PODs 3-7 total bilirubin median	124	-0.558	-1.692-0.575	0.335

Appendix 6

**Table 9:** Joint MAP-RAP model The joint model includes initial strategy, MAP, and RAP. CI, confidence interval; MAP, mean arterial pressure; OR, odds ratio; RAP, right atrial pressure.

Predictor	Scale	OR	95% CI	P
MAP	+5 mmHg	0.235	0.085-0.553	<0.001
RAP	+2 mmHg	1.009	0.657-1.575	0.968

Appendix 7

**Table 10:** Exploratory model-performance summaries AUC, area under the receiver-operating-characteristic curve. These descriptive results are not a formal test of superiority between markers. Because the analyses followed exploratory marker selection, the leave-one-out estimates do not remove all selection-related optimism and do not establish clinical validation. MAP, mean arterial pressure; MPP, mean perfusion pressure.

Assessment	Model	AUC	Brier score	Log loss
Apparent	Strategy only	0.822	0.097	0.318
Apparent	Strategy + MAP	0.891	0.079	0.264
Apparent	Strategy + MPP	0.874	0.083	0.281
Leave-one-out	Strategy only	0.718	0.103	0.34
Leave-one-out	Strategy + MAP	0.861	0.088	0.292
Leave-one-out	Strategy + MPP	0.828	0.092	0.311

Appendix 8

Appendix 9
